# Scaling nanoribbon transistors with monolayer transition metal dichalcogenides

**DOI:** 10.1038/s41565-026-02161-w

**Published:** 2026-06-02

**Authors:** Tara Peña, Anton E. O. Persson, Andrey Krayev, Áshildur Friðriksdóttir, Haotian Su, Yuan-Mau Lee, Young Suh Song, Kathryn Neilson, Zhepeng Zhang, Anh Tuan Hoang, Jerry A. Yang, Lauren Hoang, Shan X. Wang, Andrew J. Mannix, Paul C. McIntyre, Eric Pop

**Affiliations:** 1 Department of Electrical Engineering, Stanford University, Stanford, CA USA; 2 Department of Microtechnology and Nanoscience, Chalmers University of Technology, Gothenburg, Sweden; 3 HORIBA Scientific, Novato, CA USA; 4 Department of Materials Science & Engineering, Stanford University, Stanford, CA USA; 5 Geballe Laboratory for Advanced Materials, Stanford University, Stanford, CA USA; 6 Stanford Institute for Materials and Energy Sciences, SLAC National Accelerator Laboratory, Menlo Park, CA USA; 7 Stanford Synchrotron Radiation Lightsource (SSRL), SLAC National Accelerator Laboratory, Menlo Park, CA USA; 8 Precourt Institute for Energy, Stanford University, Stanford, CA USA; 9 Department of Applied Physics, Stanford University, Stanford, CA USA

**Keywords:** Electronic devices, Electrical and electronic engineering

## Abstract

Nanoscale transistors demand aggressive scaling of all channel dimensions—length, width and thickness. Two-dimensional semiconductors (2DS) provide the ultimate thickness limit, yet good device performance has largely remained restricted to micrometre-wide channels. Here we report monolayer 2DS nanoribbon transistors with both n- and p-type operation, fabricated by a top-down multipatterning process that includes ‘anchored’ contacts to limit nanoribbon delamination. This approach achieves channel lengths and widths down to 25–30 nm, with minimal edge degradation confirmed through nanoscale characterization, including tip-enhanced photoluminescence. Integrated with thin high-*κ* gate dielectrics, the devices deliver on-state currents up to 560, 420 and 130 µA µm^−1^ at a drain-to-source voltage of 1 V for n-type MoS_2_, n-type WS_2_ and p-type WSe_2_, respectively. These results exceed prior single-gated 2DS nanoribbon reports, with WS_2_ improving by more than two orders of magnitude, even for normally off (enhancement-mode) operation. Overall, these findings position top-down patterned 2DS nanoribbons as promising building blocks for future nanosheet transistor architectures.

## Abstract (web summary)

Monolayer TMD nanoribbon transistors using anchored contacts and multipatterning achieve high on-state currents down to 25 nm widths, positioning them as key building blocks for future gate-all-around nanosheet electronics.

## Main

The history of transistors for digital computing has experienced only three major changes in device architecture: the transition from bipolar to metal-oxide semiconductor field-effect transistors (FETs)^1^ in the 1970s, the transition to FinFET or tri-gate transistors^2^ around 2007, and the present transition to gate-all-around (GAA) nanosheets^3^ in 2025. Although silicon-based GAA transistors are expected to scale for at least another decade, it is unclear if the further thickness reduction required below 3 nm to retain electrostatic control is feasible due to the degradation of electrical properties^4–6^. Instead, two-dimensional (2D) semiconductors, like monolayer transition metal dichalcogenides (TMDs), are appealing alternatives due to their good electrical properties (for example, mobility and band gap) in subnanometre thin films^7,8^ and their potential in scaled GAA devices^9^. Accordingly, 2D transistors have been recently placed on technology roadmaps^10^, with targeted integration by the late 2030s or early 2040s.

The most important building block of GAA nanosheet transistors is the nanoribbon channel, which must be between 10 to 50 nm wide^11^ and atomically thin for the best electrostatic gate control^12,13^. Monolayer 2D semiconductors with subnanometre thickness should also enable shorter, sub-5 nm, gates^14,15^ than thicker silicon nanosheets^7^, making them promising for continued scaling and higher device density. However, so far, most demonstrations of good performance in monolayer 2D TMD transistors have used short, sub-100 nm, but micrometre-wide channels. The reasons are manifold but probably include difficulty in fabrication (for example, TMD delamination and lithography limitations), difficulty in making good contacts and mobility degradation due to edge imperfections. Little is known, for example, about how or if charge transport in narrow TMD ribbons changes in channel widths below a micrometre, and there are concerns about magnified edge effects in such devices^16,17^.

Here we tackle the challenges mentioned above (adhesion, width scaling, contacts and edge roughness) by realizing n- and p-type monolayer TMD nanoribbons with similar performance as co-fabricated micrometre-wide control devices. A key advance is anchoring the contacts to the substrate during fabrication, which limits nanoribbon delamination and cracking, allowing us to study many such devices. We also introduce a multipatterning approach to achieve nanoribbon widths down to 25 nm. With these advances, we reach a high current density in monolayer MoS_2_ nanoribbons, over 600 μA μm^−1^ with a SiO_2_ gate dielectric (560 μA μm^−1^ with a HfO_2_ dielectric) at a drain-to-source bias of 1 V. We also improve saturation current densities in monolayer WS_2_ nanoribbons by over 100× in enhancement mode, in normally off devices. Imaging the nanoribbons and their edges by tip-enhanced photoluminescence (TEPL) and transmission electron microscopy (TEM) suggests that edge disorder is not the limiting factor at these dimensions, indicating that such top-down monolayer TMDs are promising candidates for future nanosheet transistors.

## Fabrication of anchored nanoribbons

**Fig. 1:**
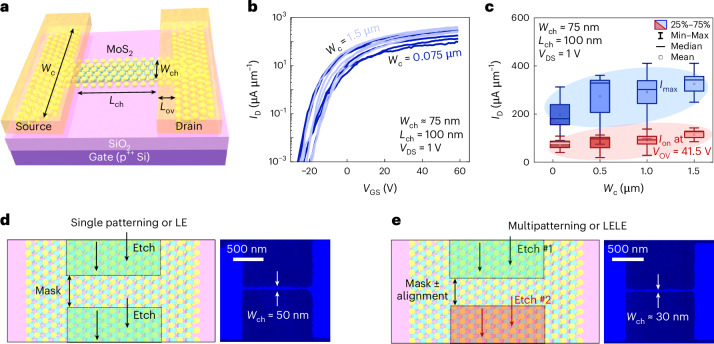
Anchored contacts and multipatterning for improved device fabrication. **a**, Schematic of the dog-bone-shaped back-gated monolayer MoS_2_ transistor, defining the channel width (*W*_ch_), length (*L*_ch_), contact width (*W*_c_) and contact overlap region (*L*_ov_). **b**, Transfer characteristics of various short-channel devices (100 nm), displaying larger contact widths in light blue (1.5 µm) and smaller contact widths in dark blue (75 nm). **c**, Box plots of the maximum drain current density (*I*_max_) and drain current at fixed gate overdrive (*I*_on_ at *V*_ov_ = *V*_GS_ – *V*_T_) for varying contact widths. Each box plot includes five to six devices, totalling 22 tested devices. **d**, Schematic of LE single patterning approach (left), which can define nanoribbon widths down to ~50 nm with reduced e-beam dose. Representative false-coloured SEM image (right) of the resulting nanoribbon. **e**, Schematic of the LELE multipatterning strategy (left; Extended Data Fig. 1 provides more details), used to achieve nanoribbon widths below 50 nm. False-coloured SEM image (right) shows a nanoribbon with ~30 nm width.

Due to the lack of out-of-plane chemical bonds, monolayer TMDs adhere to substrates by van der Waals forces, making them prone to delamination during lithography, etching and wet processing. To mitigate this, we designed a dog-bone-shaped structure in which the TMD is narrow only in the channel but expands into wider pads under the source and drain contacts (Fig. 1a). These micrometre-sized regions anchor the nanoribbon to the substrate, thereby increasing the mechanical stability and reproducibility when reducing the nanoribbon widths. The approach is somewhat analogous to the wider source and drain regions used for silicon nanosheets to enable channel release and reduce the contact resistance^18^.

We first study MoS_2_ nanoribbons on conventional SiO_2_ (96 nm) on p^++^ Si substrates, which also serve as back-gates. This allows more rapid process optimization, because the monolayer MoS_2_ is grown directly on SiO_2_, enabling higher throughput and better adhesion than layer-transferred films. As shown in Fig. 1b, short-channel devices with width *W*_ch_ ≈ 75 nm and *W*_c_ of either 75 nm or 1.5 μm display nearly identical transfer curves. Encouragingly, we observe a good maximum current density, up to *I*_max_ ≈ 400 µA µm^−1^ at *V*_DS_ = 1 V, and off-state currents limited by the measurement noise floor. Figure 1c shows that the current at fixed gate overdrive, *I*_on_, at *V*_ov_ = *V*_GS_ – *V*_T_, remains nearly unchanged, whereas *W*_c_ increases by a factor of 20 (*V*_T_ is the threshold voltage; Supplementary Fig. 1b.) We attribute this behaviour to two factors: (1) the contact–channel overlap (*L*_ov_ > 100 nm) exceeds the expected current^19^ and thermal transfer length of the contacts, and (2) a fabrication process that minimizes patterning damage at sub-100 nm widths, which we discuss below (we estimate the temperature profile along the nanoribbons in Supplementary Fig. 2). Thus, the dog-bone-shaped structure preserves the nanoribbon transistor behaviour, greatly improving our yield due to contact anchoring. Without it, sub-100 nm-wide nanoribbons often delaminate during processing, whereas the anchored contacts enabled >85% yield down to 60 nm widths (Supplementary Fig. 1c). For these reasons, the nanoribbons investigated in the remainder of the manuscript use the wider *W*_c_ = 1.5 μm, although we note that industrial manufacturing will require alternative yield improvement methods compatible with smaller contact areas^18,20^.

**Extended Data Fig. 1:**
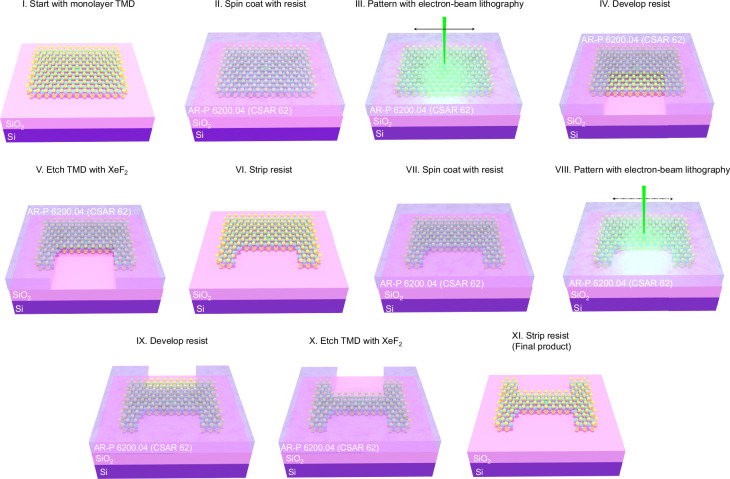
Litho-etch-litho-etch (LELE) patterning of monolayer TMD nanoribbons. The process involves two sequential e-beam lithography and etch steps. This dual-exposure approach enables sub-30 nm features by overcoming proximity-effect limitations inherent to single-step patterning, while maintaining the same (overall) low dose. See Methods for more information about doses and the low-residue resist used for lithography.

Although single-step lithography and etching (LE; Fig. 1d) is commonly used to pattern the 2D channel in academic studies, we wanted to limit the electron-beam (e-beam) exposure dose to reduce the density of lithographically induced defects^21,22^; thus, this method reaches a limit of ~50 nm widths due to the reduced dose and other fabrication trade-offs (Methods). To make narrower ribbons, we use a litho–etch–litho–etch (LELE) multipatterning approach inspired by modern industrial lithography (Fig. 1e and Extended Data Fig. 1). This enables nanoribbons down to ~25 nm widths and maintains the same overall low dose as the single patterning LE approach (Methods). Achieving sub-25 nm wide nanoribbons should be possible by optimizing the TMD adhesion and anchoring; however, sub-15 nm nanoribbons may not be desirable for GAA transistors due to larger parasitic capacitance^4,23^.

## Electrical characterization of MoS_2_ nanoribbon transistors

**Fig. 2:**
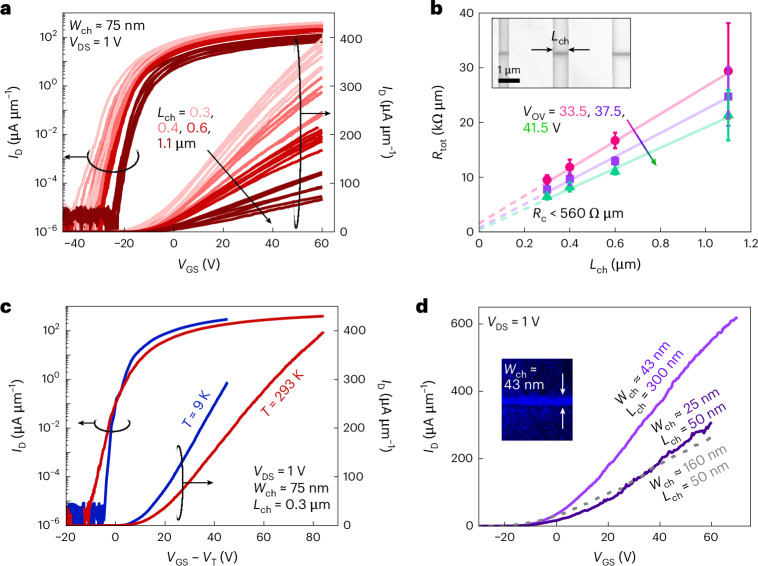
Monolayer MoS_2_ nanoribbons on SiO_2_. **a**, Transfer characteristics of nanoribbons with four different channel lengths, showing high on-state current density and consistent device behaviour. Between six and ten nanoribbons are measured for each length and 30 devices in total. *W*_ch_ = 75 nm; *W*_c_ = 1.5 μm. **b**, Transfer-length-method analysis of the same devices; each symbol and error bar represent the average and standard deviation of devices with the same channel length, respectively. The contact resistance is comparable with state-of-the-art MoS_2_/Au contacts reported^6^ in the literature (*R*_c_ < 560 Ω μm at the highest *V*_ov_). Inset: SEM image of such a transfer-length-method structure. **c**, Transfer characteristics of a nanoribbon device at low temperature (9 K) compared with room temperature, as a function of the *V*_GS_ – *V*_T_ overdrive; *V*_T_ is taken at a constant current density of 100 nA μm^−1^. **d**, Transfer characteristics of other nanoribbons, at *V*_DS_ = 1 V. The two with 43 nm (LE) and 25 nm (LELE) channel widths reach *I*_max_ ≈ 620 µA µm^−1^ and ~310 µA µm^−1^, respectively, some of the highest current densities reported so far for single-gated monolayer TMD nanoribbons at these widths. The 160 nm wide nanoribbon (dashed grey line, also on the LELE chip) has a similar current density as the 25 nm wide device, suggesting that both devices were limited by a higher contact resistance rather than edge disorder. The inset is a false-coloured SEM of the 43 nm wide nanoribbon device.

We also investigate the effect of contact resistance, by evaluating nanoribbon behaviour as a function of channel length using ten transfer-length-method structures. These nanoribbons have *W*_ch_ ≈ 75 nm and anchored contacts with *W*_c_ ≈ 1.5 μm. As shown in Fig. 2a, the devices exhibit excellent channel length dependence, maintaining stable characteristics and achieving an on-state current density up to ~400 µA µm^−1^ at channel lengths of *L*_ch_ ≈ 300 nm and *V*_DS_ = 1 V. Figure 2b displays the total device resistance *R*_tot_ versus channel length and the linear extrapolation yields a contact resistance of *R*_c_ < 560 Ω µm, or 190 ± 370 Ω µm from the linear fit at the highest *V*_ov_, comparable with some of the best MoS_2_/Au contacts reported so far^6^. We note that *R*_c_ and *R*_tot_ are normalized by the channel width *W*_ch_, not by *W*_c_, because the contacts have a non-negligible overlap with the channel (*L*_ov_ > 100 nm, greater than the expected current transfer length at these contacts^6,19^), as shown in Fig. 1a.

Interestingly, we do not see mobility degradation in nanoribbons (~75 nm wide) compared with much wider devices (~850 nm wide) fabricated on the same chip. As summarized in Supplementary Fig. 3, the field-effect electron mobility *μ*_FE_ is in the range of 30–60 cm^2^ V^−1^ s^−1^ for ten devices at room temperature, with greater device-to-device variation than any apparent width dependence. This is not surprising, because the intrinsic electron mean free path in monolayer MoS_2_ is expected^24^ to be 3–5 nm, much shorter than the nanoribbon width. Nevertheless, it is reassuring that the edges produced from the top-down patterning process used here do not appear to deteriorate device operation, a topic we return to below. Figure 2c displays the low-temperature measurements of a nanoribbon at ~9-K ambient temperature, revealing that mobility approximately doubles, which suggests that low-temperature transport is ultimately limited by impurities and possibly by the edges (Supplementary Fig. 4).

We also show transfer characteristics of other nanoribbons in Fig. 2d; one has *W*_ch_ ≈ 43 nm and *L*_ch_ ≈ 300 nm, using the single-step LE approach, and two others have *W*_ch_ ≈ 25 nm and 160 nm with *L*_ch_ ≈ 50 nm, using the LELE multipatterning approach. The first two reach *I*_max_ ≈ 620 µA µm^−1^ and ~310 µA µm^−1^, respectively, at *V*_DS_ = 1 V and similar *V*_GS_; the former is one of the highest current densities reported so far in single-gated MoS_2_ nanoribbons; the latter is one of the highest values for any monolayer ribbon of such small width. The 25 nm and 160 nm wide nanoribbons on the same LELE chip have similar current densities, suggesting that they are not limited by edge disorder. However, their *I*_max_ is lower in a shorter channel than the device on the LE chip, which we attribute to the higher contact resistance arising from our chip-to-chip variation (Supplementary Fig. 5). The nanoribbon width estimates have a 3–5 nm uncertainty (Methods), which implies 10%–20% uncertainty in current density.

## Nanoscale spectroscopic and structural characterization

**Fig. 3:**
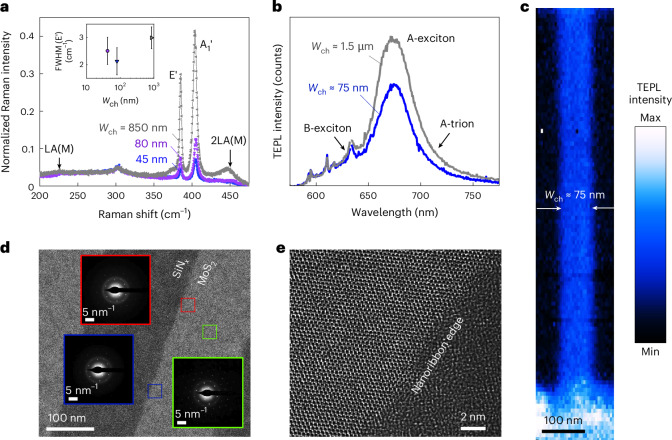
Nanoribbon material and edge characterization. **a**, Raman spectra of monolayer MoS_2_ device channels between ~45 nm to 850 nm wide, showing neither discernible broadening of the E′ or A_1_′ modes nor contributions from the LA(M) defect-mediated peak. The Raman data are normalized to the Si substrate peak at 520 cm^−1^ (not shown). Inset: full-width at half-maximum (FWHM) of the E′ mode as a function of the channel width. **b**, Averaged TEPL spectra comparing a nanoribbon channel region with the much wider anchor region of the same nanoribbon, showing minimal broadening of the A-exciton peak and a slight increase in the trion-to-exciton intensity ratio. **c**, TEPL intensity map of an entire ~75 nm wide and ~1 μm long nanoribbon, with uniform optical emission along the channel. The black spot in the top third of the nanoribbon is a measurement artefact. **d**, STEM image of two parallel monolayer MoS_2_ nanoribbons (from an array transferred onto the SiN_*x*_ membrane). Insets: diffraction patterns of three regions, revealing good crystallinity in the channel and near the edge. **e**, Magnified edge region in the TEM mode from the same STEM.

We next wish to understand the origin of good performance in our nanoribbon devices, thus we turn to materials characterization techniques to further evaluate the fabrication process and the effect of the edges. The Raman spectra shown in Fig. 3a for representative monolayer MoS_2_ devices reveal no discernible signs of damage, as the predominant E′ (in-plane) and A_1_′ (out-of-plane) phonon modes do not broaden with reduced nanoribbon widths, down to 45 nm. We do not observe defect-mediated phonon modes^25,26^ (for example, LA(M) around 227 cm^−1^) in any of our nanoribbons, suggesting that edge-related defects are not predominant. We also map our nanoribbons with TEPL (Fig. 3b,c), which reveal uniform signal across a long and narrow channel (*L*_ch_ ≈ 1 µm, *W*_ch_ ≈ 75 nm). The TEPL spectra confirm the quality of the nanoribbons, where the A-exciton does not broaden compared with the wider regions. The nanoribbons do exhibit a slightly larger trion-to-exciton ratio (Supplementary Fig. 6), which potentially suggests doping from the edges (see Extended Data Fig. 4 for more discussion).

To visualize the atomic structure and edge roughness, we performed high-angle annular dark-field scanning transmission electron microscopy (STEM) and monochromated TEM imaging on monolayer MoS_2_ nanoribbons transferred onto 10 nm thick SiN_*x*_ membranes (Fig. 3d,e). Compared with the nanoribbon transistors, these samples may be subject to some additional damage from the transfer process and the e-beam exposure during imaging. Despite this, the nanoribbon edges appear clean, with edge roughness of at most a few nanometres. The edge termination probably alternates between zigzag and armchair segments, as expected from a top-down patterning process without edge-selective anisotropy. Energy-dispersive X-ray spectroscopy and electron energy loss spectroscopy reveal no discernible accumulation of oxygen or fluorine near the edges in monolayer MoS_2_ and WSe_2_ (Supplementary Figs. 7–9). These findings suggest that our top-down fabrication approach does not introduce observable disorder or contamination of the nanoribbons and their edges, supporting the lack of degradation seen in electrical characteristics.

## High-*κ* dielectric integration

**Fig. 4:**
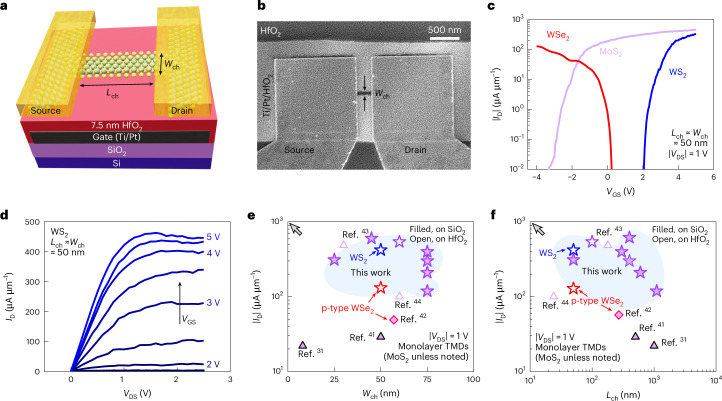
Complementary monolayer TMD nanoribbons with a high-*κ* dielectric. **a**, Schematic of a monolayer nanoribbon including anchored contacts, here with a thin HfO_2_ gate dielectric (figure not to scale). **b**, Top-down SEM image of a representative nanoribbon transistor. **c**, Measured transfer characteristics of monolayer MoS_2_, WS_2_ and WSe_2_ nanoribbons with a high-*κ* dielectric, all with channel lengths and widths of ~50 nm. **d**, Measured output characteristics of monolayer WS_2_ nanoribbon, showing well-behaved, normally off (enhancement-mode) operation with high current saturation. **e**, Comparing |*I*_max_| of single-gated monolayer TMD nanoribbons versus channel width, at |*V*_DS_| = 1 V and maximum |*V*_GS_|. Unlabelled symbols are MoS_2_, whereas WS_2_ and WSe_2_ are labelled. Our devices, marked by star symbols, reach some of the highest current densities reported so far. Our WS_2_ (blue star) has the highest *I*_max_ ≈ 420 μA μm^−1^ to date (at *V*_DS_ = 1 V) in a monolayer nanoribbon of this 2D semiconductor. Filled markers are on SiO_2_ back-gate substrates^31,39–42^; open markers are devices with a high-*κ* gate dielectric^43,44^. Symbols with the red border are p-type WSe_2_ and all others are n type. **f**, Additional benchmarking of single-gated nanoribbon devices, here versus channel length *L*_ch_. Block arrows point to the desirable corner in both benchmarking plots.

To reduce the operating voltage of our nanoribbon transistors, we integrate ultrathin high-*κ* dielectrics into the gate stack. Achieving this involves transferring monolayer TMD films onto prepatterned local back-gates with HfO_2_ dielectric (~1.5 nm equivalent oxide thickness) and then applying our optimized nanoribbon process described earlier (Methods). The schematic of the resulting nanoribbon devices is shown in Fig. 4a and confirmed by top-down scanning electron microscopy (SEM) in Fig. 4b.

Here, we expand beyond MoS_2_ by also examining monolayer n-type WS_2_ and p-type WSe_2_ nanoribbons, all patterned down to ~50 nm widths. Raman spectroscopy (Supplementary Fig. 10) indicates that the nanoribbon fabrication steps do not appear to introduce additional damage. Figure 4c compares the transfer characteristics of three such nanoribbon channels that are just 50 nm × 50 nm. Among these, monolayer MoS_2_ achieves the highest on-state current density, *I*_max_ ≈ 460 µA µm^−1^ at *V*_DS_ = 1 V, although with a negative *V*_T_ (that is, a normally on, depletion-mode device). An ~60 nm wide monolayer MoS_2_ nanoribbon reaches *I*_max_ ≈ 560 µA µm^−1^ at *V*_DS_ = 1 V (Supplementary Fig. 11).

On the other hand, monolayer WS_2_ nanoribbons show a desirable positive *V*_T_ (that is, a normally off, enhancement-mode device), but still reach *I*_max_ ≈ 420 µA µm^−1^ at *V*_DS_ = 1 V (and up to ~460 μA µm^−1^ at *V*_DS_ = 1.5 V; Fig. 4d). These WS_2_ current densities are some of the highest values reported so far, by >100×, for a nanoribbon of this material, probably due to our fabrication process and our use of stressed Ni/Au contacts^27^ with good *R*_c_ ≈ 675 ± 268 Ω µm (Supplementary Fig. 12). Finally, the p-type WSe_2_ nanoribbons reach |*I*_max_| ≈ 130 µA µm^−1^, an encouraging result given its desirable negative *V*_T_ (that is, a normally off, enhancement-mode device) and the historical performance gap between p-type and n-type TMD transistors. Figure 4d shows the output curves of the 50 nm × 50 nm WS_2_ nanoribbon, with good current saturation and device turn-off at zero gate voltage, essential for future circuit implementation.

**Extended Data Fig. 2:**
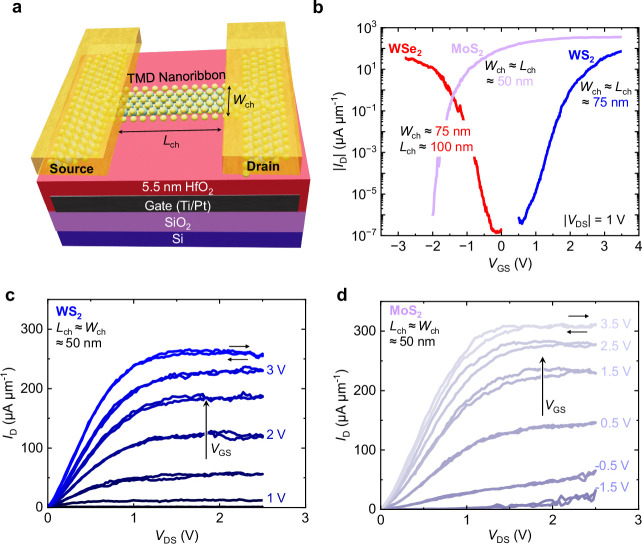
Off-state current and output characteristics for various monolayer transition metal dichalcogenide (TMD) nanoribbons on 5.5 nm HfO_2_ back-gate dielectrics. **a**, Device schematic, for various TMD nanoribbons with Au contacts on 5.5 nm HfO_2_ back-gate dielectric. Note that the HfO_2_ dielectric on these test chips was slightly thinner than the 7.5 nm thick HfO_2_ used in Fig. 4 of the main text. **b**, Measured transfer characteristics of monolayer MoS_2_, WS_2_, and WSe_2_ nanoribbons with Au source and drain contacts, on local back-gates with high-κ dielectric, including the deep off-state. Note that Au source-drain contacts are optimal for MoS_2_ while Ni/Au and Pd/Au are preferable for WS_2_ and WSe_2_, respectively, explaining the lower on-current densities seen here compared to Fig. 4c in the main text. Nevertheless, these each display *I*_max_/*I*_min_ ratios over 10^8^. **c**, Output characteristics of a WS_2_ nanoribbon with dimensions and voltages as listed. **d**, Output characteristics of a MoS_2_ nanoribbon with dimensions and voltages as listed. Both nanoribbons here had Au source and drain contacts, for direct comparison. (In contrast, WS_2_ nanoribbons in main text Fig. 4 had Ni/Au contacts.) Small arrows indicate voltage sweep directions, illustrating small hysteresis. All measurements are at room temperature.

**Extended Data Fig. 3:**
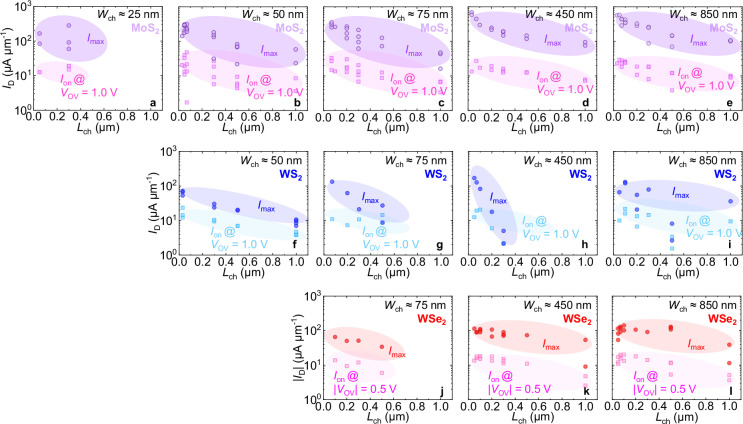
Width-dependent current density across our monolayer TMDs. Drain current density (*I*_D_) vs. channel length (*L*_ch_) for monolayer n-type MoS_2_ devices with channel widths (*W*_ch_) of approximately **a**, 25 nm, **b**, 50 nm, **c**, 75 nm, **d**, 450 nm, and **e**, 850 nm. Corresponding data for monolayer n-type WS_2_ with *W*_ch_ of approximately **f**, 50 nm, **g**, 75 nm, **h**, 450 nm, and **i**, 850 nm. Corresponding data for monolayer p-type WSe_2_ with *W*_ch_ of approximately **j**, 75 nm, **k**, 450 nm, and **l**, 850 nm. All devices in this figure have Au drain and source contacts, and were fabricated on 5.5 nm HfO_2_ with local back-gates (see Extended Data Fig. 2a), measured at |*V*_DS_ | = 1 V. Each panel shows both *I*_max_ (at maximum |*V*_GS_ | = 3 V for WSe_2_ and 3.5 V for the others) and *I*_on_ at *V*_ov_ = |*V*_GS_ – *V*_T,cc_ | = 1 and 0.5 V, for n-type and p-type films respectively, where *V*_T,cc_ is taken at *I*_D_ = 100 nA μm^−1^. Within the device-to-device variability typical of academic nanofabrication, we observe no obvious changes in current density between nanoribbons (left columns) and microribbons (right columns) of a given material, after accounting for *V*_T_ changes. The slightly higher *I*_max_ in wider channels of MoS_2_ and WS_2_ is due to their lower *V*_T,cc_ (see Extended Data Fig. 4 for more discussion); accordingly, no obvious nano- vs. microribbon difference is seen in *I*_on_. Note, some nanoribbon widths are missing for WS_2_ and WSe_2_ due to adhesion and yield issues from the layer transfer process onto HfO_2_ with local back-gates.

**Extended Data Fig. 4:**
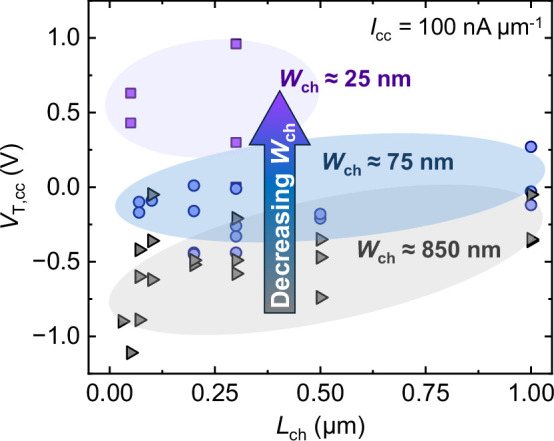
Width-dependent threshold voltage across monolayer MoS_2_ nanoribbons. Threshold voltage as a function of channel length and width, for our monolayer MoS_2_ devices on HfO_2_ from Extended Data Fig. 3a–e. Displayed are devices with channel widths *W*_ch_ ≈ 850 nm (grey triangles), 75 nm (blue circles), and 25 nm (purple squares). *V*_T,cc_ is estimated at a constant current of 100 nA µm^−1^. *V*_T,cc_ increases as *W*_ch_ is reduced, causing an apparent decrease of *I*_max_ for the nanoribbons at maximum *V*_GS_ ( = 3.5 V here on ~5.5 nm HfO_2_ back-gate dielectric). Our findings here are consistent with earlier work^49^ on thick MoS_2_ nanoribbons (~10 layers), which also found increased *V*_T_ as *W*_ch_ is reduced, attributing it to negative charges adsorbed at the edges. Fixed edge charges may arise from fabrication or atmospheric exposure, and are likely below electron energy loss spectroscopy detection limits. On thick back-gate dielectrics (comparable to or thicker than *W*_ch_), the relatively higher contribution of fringing fields at the edges could have a competing effect and *decrease V*_T_ as *W*_ch_ is reduced, as seen in Supplementary Fig. 3c for devices on 96 nm SiO_2_. The analysis here was conducted for the forward-sweep of the transfer curves, however, we note that the hysteresis was quite small for these devices (see Extended Data Fig. 2d), thus rendering the trends here to be robust to either sweep direction.

Figure 4e,f compares our results with other single-gate monolayer TMD nanoribbons reported to date, at |*V*_DS_| = 1 V. Here we benchmark the current density, |*I*_max_|, rather than mobility or contact resistance, because *I*_max_ is less prone to measurement error (its greatest uncertainty comes from the nanoribbon width) and because, in principle, the threshold voltage can be adjusted by gate-stack engineering^28,29^. *I*_max_ also incorporates information about contact resistance and mobility, and it is ultimately responsible for circuit delays, which are inversely proportional to the current density^30^. Our monolayer MoS_2_ nanoribbons match or exceed previously reported *I*_max_ at comparable channel widths, whereas our monolayer WS_2_ and WSe_2_ nanoribbons exceed existing results, the WS_2_ by a factor of >100× (we provide additional output curves, current drive statistics and *V*_T_ discussion across 140 devices; Extended Data Figs. 2–4). Although sub 50 nm and even sub-10 nm-wide nanoribbons (monolayer and multilayer) have been demonstrated using bottom-up^31–35^ or anisotropic etching techniques^36^, such devices have not reached the current densities of our top-down patterned nanoribbons at comparable channel widths.

**Extended Data Fig. 5:**
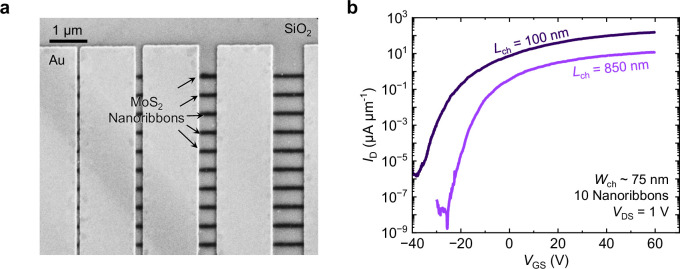
Electrical performance of monolayer MoS_2_ nanoribbon arrays. **a**, Top-down scanning electron image of the nanoribbon array, showing widths down to ~75 nm and 10 parallel nanoribbons. The devices here are monolayer MoS_2_ directly grown and fabricated onto 96 nm SiO_2_ on *p*^++^ Si. In the image, the nanoribbons appear dark, while the lighter large rectangles are the Au contacts. **b**, Measured transfer characteristics of short- and long-channel nanoribbon array devices. The on-state current density is comparable to that of single nanoribbons (see main text Fig. 2a). The off-state current density is one to two orders of magnitude lower than that measured in individual nanoribbons due to the width difference and differences in measurement settings, and comparable to some of the best-known micrometer-scale devices (*I*_max_/*I*_min_ current ratio > 10^9^). The short-channel device (100 nm here) exhibits slightly degraded off-state, likely from *V*_T_ roll-off and other short-channel effects, on the thicker SiO_2_ (96 nm) back-gate oxide.

While we have shown that the on-state of these nanoribbons can be as good as existing micrometre-scale devices, a remaining aspect is to enquire whether the off-state is affected by their edges. To probe this regime, we measured arrays of MoS_2_ nanoribbons (Extended Data Fig. 5). These reach *I*_max_/*I*_min_ current ratios of >10^9^, limited by the measurement noise floor, also comparable with some of the best-known micrometre-scale devices reported to date. In other words, we conclude that at least down to the channel widths probed here, there is no measurable edge conduction in the off-state, probably due to the mixed character^16,37^ of our edges (Fig. 3e). To our knowledge, no previous efforts on parallel nanoribbon arrays have probed the deep off state, although a previous study^38^ on random networks composed of narrower (10–30 nm) ribbons also found no evidence of off-state degradation. We provide additional comparisons of monolayer TMD nanoribbons, including some with unconventional geometry or fabrication approaches in Supplementary Table 1.

## Conclusions

We have demonstrated both n- and p-type (that is, complementary) nanoribbon transistors with MoS_2_, WS_2_ and WSe_2_ at the ultimate limit of monolayer channel thinness. These achieve high current densities in channels down to ~25 nm widths, with WS_2_ nanoribbons in particular showing desirable, normally off (enhancement-mode) behaviour, with good current saturation (~460 μA μm^−1^ at 1.5 V drain-to-source voltage). The nanoribbons were enabled by mechanically robust anchored contacts that improve yield, a low-dose multipatterning strategy with low-residue resist, and minimal edge degradation, studied by advanced nanoscale imaging, including TEPL. Our work shows that scaling monolayer TMD channels down to ~25 nm widths does not degrade their on- or off-state, and such nanoribbon demonstrations are more technologically relevant than micrometre-wide devices. Looking ahead, the compatibility with high-*κ* dielectrics, complementary device polarity and good performance across several TMDs position monolayer nanoribbons as important building blocks of future GAA^9^ nanosheet transistors.

## Methods

### TMD synthesis and transfer

All monolayer MoS_2_ and WS_2_ films were grown onto thermal 96nm SiO_2_/Si and sapphire substrates, respectively, as described in previous works^45,46^. The chemical vapour deposition grown monolayer WSe_2_ on sapphire was purchased from 2D Semiconductors. For devices on HfO_2_ (either 5.5 nm or 7.5 nm thick) with local back-gates, all the monolayer films were transferred from their growth substrates by spinning polystyrene (PS) at 1,250 rpm for 60 s onto the growth substrate, followed by baking at 85 °C for 5 min. The TMD/PS stack was then immersed in deionized water to delaminate it from the growth substrate. The TMD/PS stack was then placed onto the chip with local back-gate structures, dried with N_2_ and left in a N_2_ dry box overnight. The following morning, the chips were heated at 85 °C for 1 h, 150 °C for 1 h and then cooled to room temperature. The chips were then placed in toluene overnight, followed by acetone and isopropanol cleaning (10 min each) to remove the PS from the surface. Before device fabrication, the chips were annealed in vacuum (~10^−5^ torr) at 200 °C for 2 h to promote TMD adhesion to HfO_2_.

### Fabrication process

For the devices shown in Figs. 1, 2 and 3a, the monolayer MoS_2_ films were grown by solid-source chemical vapour deposition directly onto dry thermal SiO_2_ (96 nm) on p^++^ Si (resistivity, <5 mΩ cm) substrates and were processed with no transfer, thereby having better adhesion and higher yield. For these devices, e-beam lithography (Raith EBPG 5200+ with 100 kV accelerating voltage) was used to first define coarse probing pads, which consist of e-beam-evaporated SiO_2_ (20 nm)/Ti (1 nm)/Pt (15 nm), where SiO_2_ is used to limit probing pad leakage to the Si back-gate. To construct the local back-gate samples (Fig. 4a–d), back-gate metals of Ti (1 nm)/Pt (13 nm) are defined by lift-off and then 7.5 nm HfO_2_ is deposited by atomic layer deposition at 200 °C.

All nanoribbon channels were then defined using e-beam lithography and etched using XeF_2_. A high-resolution AR-P 6200.04 (CSAR 62) resist was spun (3,000 rpm, 60 s) and baked at 120 °C (5 min), yielding an ~50 nm resist thickness. Compared with conventional polymethyl methacrylate recipes, we found that the CSAR 62 resist allows for lower writing doses (here we use 425–475 µC cm^−2^) and leaves less residue on the TMD surface. The resist was developed in room-temperature xylenes (45 s), followed by a quick isopropanol dip. This procedure would be repeated if implementing the multipatterning (or LELE) approach (Extended Data Fig. 1). We found that a colder or more dilute developer can further improve resolution (even using the single patterning (LE) process), but at the cost of requiring higher doses that risk TMD damage^21,22^. After channel formation, we defined fine source/drain contacts by a third e-beam lithography step and deposited using e-beam evaporation (~10^−8^ torr). For monolayer MoS_2_, ~40 nm Au contacts are used, without an adhesion layer^6^. For monolayer WS_2_, stressed Ni (10 nm)/Au (20 nm) fine contacts were used, following our previous work^27^, to obtain good *R*_c_. For monolayer WSe_2_, Pd (10 nm)/Au (20 nm) contacts are utilized to promote hole injection, given the higher work function of Pd. Monolayer WSe_2_ devices in this work were immersed in chloroform overnight before measurements, as chloroform has been found to lower *R*_c_ for holes^47^ in monolayer WSe_2_. Extended Data Figs. 2–4 show all three monolayer TMDs with Au (35 nm) contacts and with a 5.5 nm HfO_2_ back-gate dielectric, where both the n-type semiconductors experience vacuum annealing and the p-type semiconductor is immersed in chloroform overnight before measurements.

### Electrical measurements

Unless otherwise stated, electrical measurements were performed at room temperature using a Janis ST-100 vacuum probe station at ~10^−4^ torr, using a Keithley 4200A semiconductor parameter analyser. All monolayer MoS_2_ devices presented in this work were first annealed at 250 °C for 2 h under vacuum inside the probe station, to improve *R*_c_ and remove adsorbates from the channel^6^. The monolayer MoS_2_ devices are then measured after cooling back to room temperature, without breaking vacuum. The monolayer WS_2_ devices shown in Fig. 4 did not undergo any annealing procedures (to retain the strain state from the Ni/Au contacts), whereas the WS_2_ devices (Extended Data Figs. 2 and 3) experienced annealing to improve their Au contacts. All monolayer WSe_2_ devices presented here did not experience any annealing before electrical testing in vacuum. Cryogenic measurements were conducted using a Lakeshore cryoprobe station under vacuum (~10^−6^ torr) and a Keithley 4200A semiconductor parameter analyser.

### Material characterization

Micro-Raman spectroscopy was performed using a Horiba LabRAM instrument with a 532 nm laser with 1,800 spectrometer grating at a laser power of 120 µW. Measurements were performed at room temperature and in ambient conditions. Atomic force microscopy (AFM) was conducted using a Bruker Dimension Icon in the standard tapping mode with a NSC18 Pt probe. Scanning electron microscopy (SEM) was performed using either an FEI Magellan or Helios, with an accelerating voltage of 2 kV and beam current of 43 pA.

After the nanoribbons were visualized by AFM and/or SEM, we extracted the average nanoribbon width across the channel. This channel extraction was systematically conducted using ImageJ analysis software in which an average contrast line profile across the substrate and nanoribbon can be determined. This contrast line profile can be fitted to a Gaussian function in which the nanoribbon width is defined as the full-width at half-maximum of the fitted function. The fitting allows us to simultaneously calculate the uncertainty in our channel width estimates, which was typically 3–5 nm.

High-resolution TEM images were taken at 80 kV using a Thermo Fisher Spectra 300 operated in the monochromated TEM imaging mode. To account for sample drift during acquisition, 80 frames were collected with drift correction enabled. The frames were then aligned and averaged to compensate for drift and improve the signal-to-noise ratio. Imaging was performed using a collection angle of 228 mrad. Final images were processed using a drift-corrected frame integration routine on the Ceta camera, which registers and integrates the aligned frames. A radial Wiener filter was applied to further suppress high-frequency noise and enhance image clarity. Energy-dispersive X-ray spectroscopy was performed in the STEM mode at 80 kV on the same instrument. Electron energy loss spectroscopy measurements were performed in the single-mode high-quality acquisition using the energy-filtered charge-coupled device detector. The microscope was operated with a 50 µm C2 aperture, corresponding to a convergence semi-angle of 21.4 mrad and a camera length of 29 mm. The beam current was set to 0.213 nA. A smaller entrance aperture was used to improve the energy resolution. The zero-loss peak was aligned before acquisition, and the spectrometer was tuned for optimal focus. The final zero-loss peak full-width at half-maximum was 1.05 eV, confirming good energy resolution. The dispersion was set to 0.3 eV per channel, suitable for resolving the C–K, N–K, O–K and F–K edges and maintaining sufficient signal intensity. The monolayer MoS_2_ samples for this experiment (Fig. 3d,e) were grown directly onto 300 nm SiO_2_ on Si substrates, and then underwent e-beam lithography and dry etching (following the channel definition procedure described above) to produce nanoribbons. The finalized nanoribbons were delaminated using a droplet of deionized water onto a polydimethylsiloxane stamp. The nanoribbons were then dry transferred from the stamp down onto a 10 nm thick SiN_*x*_ TEM window (Norcada TA301Z).

TEPL maps were collected on a LabRAM-Nano AFM Raman system (HORIBA Scientific) modified for the concurrent excitation and collection using two lasers simultaneously^48^. Excitation and collection of the Raman signal were done using the side 100×, 0.7-numerical-aperture objective (Mitutoyo) inclined at 25° to the plane of the sample. Laser power on the sample for both 633 nm and 594 nm excitations were about 150 µW. The TEPL maps were collected using Omni-Access-NC-Au (APPNano) TERS probes in the DualSpec version of the SpecTop mode where in each pixel of the map, the spectra were collected with the tip in direct contact with the sample (near + far field) and in the tapping operation with an amplitude of about 20 nm (far field). The far-field data were subtracted from the combined map to produce the pure near-field response. The monolayer MoS_2_ samples for this experiment (Fig. 3b,c) were grown directly onto 300 nm SiO_2_ on Si substrates, which then underwent e-beam lithography and dry etching (following the channel definition procedure described above) to complete the nanoribbons. The samples were vacuum annealed at ~10^−4^ torr at 250 °C for 8 h to remove the adsorbates and resist residues before experiments.

## Online content

Any methods, additional references, Nature Portfolio reporting summaries, source data, extended data, supplementary information, acknowledgements, peer review information; details of author contributions and competing interests; and statements of data and code availability are available at 10.1038/s41565-026-02161-w.

## Supplementary information

Supplementary Information Supplementary Sections 1–13, Figs. 1–12, Table 1 and references.

## Data Availability

All data needed to evaluate the conclusions in this paper are present in the Article or its Supplementary Information.

## References

[1] Kahng, D. Electric field controlled semiconductor device. US patent 3,102,230 (1963).

[2] Auth, C. et al. A 22 nm high performance and low-power CMOS technology featuring fully-depleted tri-gate transistors, self-aligned contacts and high density MIM capacitors. In *Proc. Symposium on VLSI Technology* 131–132 (IEEE, 2012).

[3] Yeap, G. et al. 2 nm platform technology featuring energy-efficient nanosheet transistors and interconnects co-optimized with 3DIC for AI, HPC and mobile SoC applications. In *Proc. IEEE International Electron Devices Meeting* 1091–1094 (IEEE, 2024).

[4] Cao, W. et al. The future transistors. *Nature* **620**, 501–515 (2023). 37587295 10.1038/s41586-023-06145-x

[5] Agrawal, A. et al. Silicon RibbonFET CMOS at 6 nm gate length. In *Proc. IEEE International Electron Devices Meeting* 605–608 (IEEE, 2024).

[6] English, C. D., Shine, G., Dorgan, V. E., Saraswat, K. C. & Pop, E. Improved contacts to MoS_2_ transistors by ultra-high vacuum metal deposition. *Nano Lett.* **16**, 3824–3830 (2016). 27232636 10.1021/acs.nanolett.6b01309

[7] O’Brien, K. P. et al. Process integration and future outlook of 2D transistors. *Nat. Commun.* **14**, 6400 (2023). 37828036 10.1038/s41467-023-41779-5 PMC10570266

[8] Liu, Y. et al. Promises and prospects of two-dimensional transistors. *Nature* **591**, 43–53 (2021). 33658691 10.1038/s41586-021-03339-z

[9] Mortelmans, W. et al. Gate oxide module development for scaled GAA 2D FETs enabling SS <75 mV dec^−1^ and record Idmax >900 μA μm^−1^ at Lg <50 nm. In *Proc. IEEE International Electron Devices Meeting* 365–368 (IEEE, 2024).

[10] Lockhart de la Rosa, C. J. & Kar, G. S. Introducing 2D-material based devices in the logic scaling roadmap. *Semicond. Dig.* **6**, 17–21 (2024).

[11] IEEE International Roadmap for Devices and Systems; https://irds.ieee.org/ (accessed 25 April 2025).

[12] Pal, A., Chavan, T., Jabbour, J., Cao, W. & Banerjee, K. Three-dimensional transistors with two-dimensional semiconductors for future CMOS scaling. *Nat. Electron.* **7**, 1147–1157 (2024).

[13] Dubey, P. K. et al. Simulation of vertically stacked 2-D nanosheet FETs. *IEEE Trans. Electron Devices* **72**, 1494–1500 (2025).

[14] Wu, F. et al. Vertical MoS_2_ transistors with sub-1-nm gate lengths. *Nature* **603**, 259–264 (2022). 35264756 10.1038/s41586-021-04323-3

[15] Chen, S. et al. Channel and contact length scaling of two-dimensional transistors using composite metal electrodes. *Nat. Electron.* **8**, 394–402 (2025).

[16] Pan, H. & Zhang, Y.-W. Edge-dependent structural, electronic and magnetic properties of MoS_2_ nanoribbons. *J. Mater. Chem.* **22**, 7280–7290 (2012).

[17] Li, Y., Zhou, Z., Zhang, S. & Chen, Z. MoS_2_ nanoribbons: high stability and unusual electronic and magnetic properties. *J. Am. Chem. Soc.* **130**, 16739–16744 (2008). 19554733 10.1021/ja805545x

[18] Mochizuki, S. et al. Stacked gate-all-around nanosheet pFET with highly compressive strained Si_1–x_Ge_x_ channel. In *Proc. IEEE International Electron Devices Meeting* 19–22 (IEEE, 2020).

[19] McClellan, C. J., Yalon, E., Smithe, K. K. H., Suryavanshi, S. V. & Pop, E. High current density in monolayer MoS_2_ doped by AlO_*x*_. *ACS Nano* **15**, 1587–1596 (2021). 33405894 10.1021/acsnano.0c09078

[20] Bourjot, E. et al. Wrap-all-around contact for nanosheet-FET and method of forming same. US patent 10,559,656 (2020).

[21] Wu, Z. et al. Defects as a factor limiting carrier mobility in WSe_2_: a spectroscopic investigation. *Nano Res.* **9**, 3622–3631 (2016).

[22] Neilson, K. *Advancing Two-dimensional Semiconductor Devices Through Growth, Fabrication, And Contact Engineering*. PhD thesis, Stanford Univ. (2025).

[23] Ahmed, Z. et al. Introducing 2D-FETs in device scaling roadmap using DTCO. In *Proc. IEEE International Electron Devices Meeting (IEDM)* 465–468 (IEEE, 2020).

[24] Wang, M. A. & Pop, E. Monte Carlo simulation of electrical transport with joule heating and strain in monolayer MoS_2_ devices. *Nano Lett.* **25**, 6841–6847 (2025). 40237296 10.1021/acs.nanolett.4c05254

[25] Mignuzzi, S. et al. Effect of disorder on Raman scattering of single-layer MoS_2_. *Phys. Rev. B* **91**, 195411 (2015).

[26] Wu, J.-B. et al. Monolayer molybdenum disulfide nanoribbons with high optical anisotropy. *Adv. Opt. Mater.* **4**, 756–762 (2016).

[27] Hoang, L. et al. Understanding the impact of contact-induced strain on the electrical performance of monolayer WS_2_ transistors. *Nano Lett.* **24**, 12768–12774 (2024). 39365938 10.1021/acs.nanolett.4c02616 PMC11488502

[28] Ko, J.-S. et al. Achieving 1-nm-scale equivalent oxide thickness top-gate dielectric on monolayer transition metal dichalcogenide transistors with CMOS-friendly approaches. *IEEE Trans. Electron Devices* **72**, 1514–1519 (2025).

[29] Ko, J.-S. et al. Sub-nanometer equivalent oxide thickness and threshold voltage control enabled by silicon seed layer on monolayer MoS_2_ transistors. *Nano Lett.* **25**, 2587–2593 (2025). 39902956 10.1021/acs.nanolett.4c01775

[30] Salman, E. & Friedman, E. G. *High Performance Integrated Circuit Design* (McGraw-Hill, 2012).

[31] Li, X. et al. Width-dependent continuous growth of atomically thin quantum nanoribbons from nanoalloy seeds in chalcogen vapor. *Nat. Commun.* **15**, 10080 (2024). 39572579 10.1038/s41467-024-54413-9 PMC11582360

[32] Saunders, A. P. et al. Direct exfoliation of nanoribbons from bulk van der Waals crystals. *Small* **20**, 2470348 (2024). 10.1002/smll.202403504 39140377

[33] Li, X. et al. Nickel particle–enabled width-controlled growth of bilayer molybdenum disulfide nanoribbons. *Sci. Adv.* **7**, eabk1892 (2021). 34890223 10.1126/sciadv.abk1892 PMC8664269

[34] Chowdhury, T. et al. Substrate-directed synthesis of MoS_2_ nanocrystals with tunable dimensionality and optical properties. *Nat. Nanotechnol.* **15**, 29–34 (2020). 31740793 10.1038/s41565-019-0571-2

[35] Ma, Z. et al. Lattice-guided growth of dense arrays of aligned transition metal dichalcogenide nanoribbons with high catalytic reactivity. *Sci. Adv.* **11**, eadr8046 (2025). 39772681 10.1126/sciadv.adr8046 PMC11708881

[36] Hoque, M. A. et al. Ultranarrow semiconductor WS_2_ nanoribbon field-effect transistors. *Nano Lett.* **25**, 1750–1757 (2025). 39846459 10.1021/acs.nanolett.4c01076 PMC11803707

[37] Davelou, D., Kopidakis, G., Kaxiras, E. & Remediakis, I. N. Nanoribbon edges of transition-metal dichalcogenides: stability and electronic properties. *Phys. Rev. B* **96**, 165436 (2017).

[38] Aslam, M. A. et al. Single-crystalline nanoribbon network field effect transistors from arbitrary two-dimensional materials. *npj 2D Mater. Appl.* **6**, 76 (2022).

[39] Chen, S. et al. Monolayer MoS_2_ nanoribbon transistors fabricated by scanning probe lithography. *Nano Lett.* **19**, 2092–2098 (2019). 30808165 10.1021/acs.nanolett.9b00271

[40] Jiang, J. et al. Schottky-barrier quantum well in two-dimensional semiconductor nanotransistors. *Mater. Today Phys.* **15**, 100275 (2020).

[41] Kotekar-Patil, D., Deng, J., Wong, S. L., Lau, C. S. & Goh, K. E. J. Single layer MoS_2_ nanoribbon field effect transistor. *Appl. Phys. Lett.* **114**, 013508 (2019).

[42] Chen, S., Zhang, Y., King, W. P., Bashir, R. & van der Zande, A. M. Edge-passivated monolayer WSe_2_ nanoribbon transistors. *Adv. Mater.* **36**, 2313694 (2024). 10.1002/adma.202313694 PMC11436303 39023387

[43] Lan, H.-Y. et al. Reliability of high-performance monolayer MoS_2_ transistors on scaled high-*κ* HfO_2_. *npj 2D Mater. Appl.* **9**, 5 (2025).

[44] O’Brien, K. P. et al. Advancing 2D monolayer CMOS through contact, channel and interface engineering. In *Proc. IEEE International Electron Devices Meeting* 163–166 (IEEE, 2021).

[45] Smithe, K. K. H., Suryavanshi, S. V., Muñoz Rojo, M., Tedjarati, A. D. & Pop, E. Low variability in synthetic monolayer MoS_2_ devices. *ACS Nano* **11**, 8456–8463 (2017). 28697304 10.1021/acsnano.7b04100

[46] Zhang, Z. et al. Chemically tailored growth of 2D semiconductors via hybrid metal–organic chemical vapor deposition. *ACS Nano* **18**, 25414–25424 (2024). 39230253 10.1021/acsnano.4c02164 PMC11412230

[47] Hoang, L. et al. Low resistance p-type contacts to monolayer WSe_2_ through chlorinated solvent doping. *Nat. Commun.* **17**, 718 (2026). 41559030 10.1038/s41467-025-65604-3 PMC12820234

[48] Krayev, A. et al. Excitation laser energy dependence of the gap-mode TERS spectra of WS_2_ and MoS_2_ on silver. *ACS Photonics* **12**, 1535–1544 (2025).

[49] Liu, H., Gu, J. & Ye, P. D. MoS_2_ nanoribbon transistors: transition from depletion mode to enhancement mode by channel-width trimming. *IEEE Electron Device Lett.* **33**, 1273–1275 (2012).

