# Supplementary material for: Scaling nanoribbon transistors with monolayer transition metal dichalcogenides
Source: Nat Nanotechnol. 2026 Jun 2;21(6):803–9. doi: 10.1038/s41565-026-02161-w (PMC13293853; doi:10.1038/s41565-026-02161-w)
Supplement: Supplementary file 1 — Supplementary Sections 1–13, Figs. 1–12, Table 1 and references. [file 41565_2026_2161_MOESM1_ESM.pdf]

nature nanotechnology

Supplementary information https://doi.org/10.1038/s41565-026-02161-w

Scaling nanoribbon transistors with monolayer transition metal dichalcogenides

In the format provided by the authors and unedited

Table of Contents

1. Contact width dependence
2. Thermal analysis of select nanoribbons
3. Nanoribbon vs. microribbon monolayer MoS_2 performance comparison
4. Low-temperature electrical measurements of monolayer MoS_2 nanoribbons on SiO_2
5. Nanoribbon width dependence and hysteresis on multi-patterning (LELE) chip
6. Tip enhanced photoluminescence (TEPL) excitation dependence
7. Energy dispersive X-ray spectroscopy (EDS) and electron energy loss spectroscopy (EELS) at a monolayer MoS_2 nanoribbon corner
8. TEPL and EELS analysis on monolayer WSe_2
9. High-angle annular dark-field (HAADF) scanning transmission electron microscopy
10. Micro-Raman spectroscopy on monolayer WS_2 and WSe_2 nanoribbons
11. Output current-voltage characteristics for monolayer MoS_2 nanoribbon on HfO_2
12. Contact resistance estimate for monolayer WS_2 nanoribbons and comparison to microribbons
13. Benchmarking table of monolayer nanoribbon transistors

1. Contact width dependence

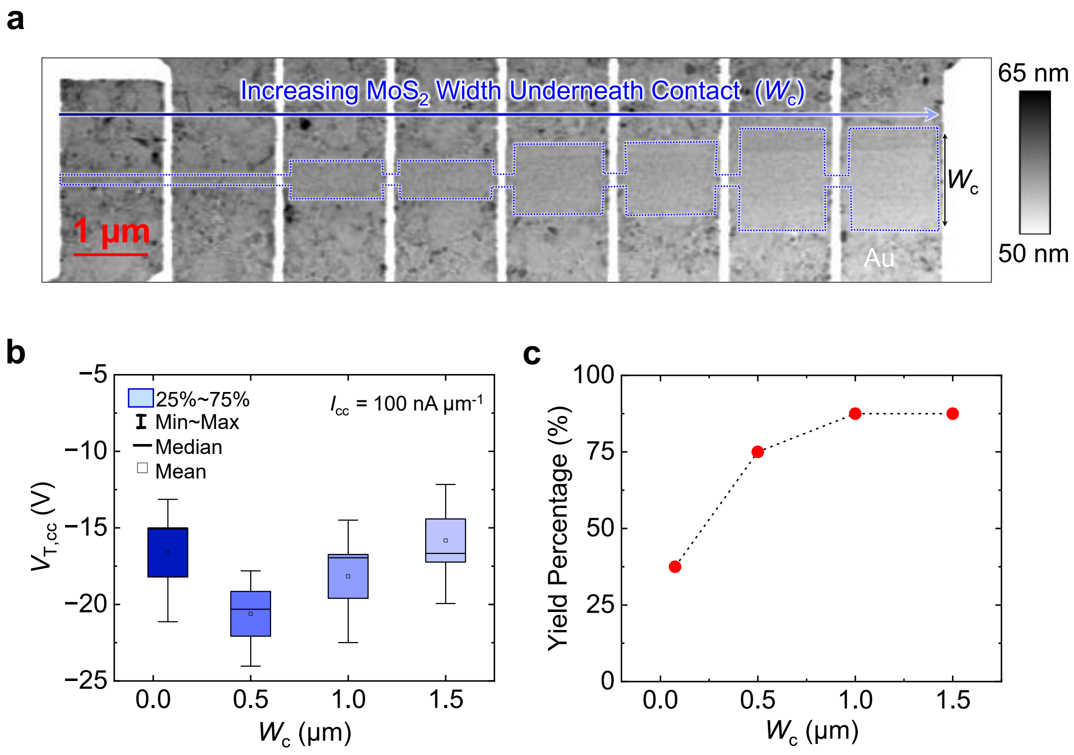

Supplementary Figure 1 | Contact width dependence of monolayer MoS_2 nanoribbon devices. a, Atomic force microscope scan of devices for the contact-width-dependent study on a 96 nm SiO_2 back-gate insulator. Here, only the nanoribbon width is scaled underneath the contacts, from $W_c \approx 75$ nm to 1.5 μm (see blue dotted outline), while the channel width (W_{ch}) and channel length (L_{ch}) are fixed to 75 nm and 100 nm respectively. Note that contacts are the same width in pairs, *i.e.* first two are 75 nm wide (same as nanoribbon channel width, W_{ch}), the next two are 500 nm, and so on. The height or z-axis scale is leveled to emphasize the contact regions, where the Au grains can be visually identified, and the smooth regions indicate where the monolayer MoS_2 is underneath. **b**, Box plot of threshold voltage ($V_{T,cc}$) for the transfer characteristics with varying contact widths (W_c) in main text **Fig. 1b**, extracted at a constant current[1] of I_D = 100 nA μm^{-1}. Each box plot consists of five to six devices. The middle solid line denotes the median value, upper and lower box ranges indicate the higher and lower quartiles, then the upper and lower whiskers highlight the maximum and minimum values. **c,** Yield curve as a function of W_c, for nanoribbons with a $W_{ch} \approx 60$ nm. Here, we employed scanning electron microscopy to visualize the nanoribbons, then documented samples with cracks and delamination as failures. Unless otherwise stated, we use W_c = 1.5 μm for our devices in the rest of the manuscript, because they had the highest yield. We caution that yield in our academic facilities is less indicative of yield in an industrial process, but rather an indication of our ease of fabrication and ability to test multiple (*e.g.,* dozens or hundreds of) devices.

2. Thermal analysis of select nanoribbons

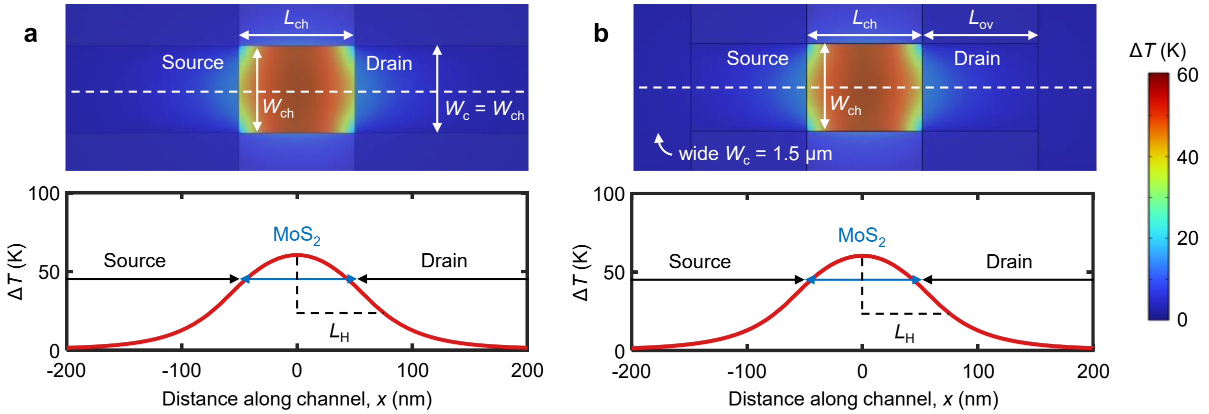

Supplementary Figure 2 | Thermal simulation of monolayer MoS_2 nanoribbon. a, Computed temperature rise (ΔT) for nanoribbon with L_{ch} = 100 nm, W_{ch} = 75 nm on 96 nm SiO_2 thickness, using 'narrow' contact regions, $W_c = W_{ch}$ = 75 nm. **b,** Same nanoribbon geometry, but using 'wide' contact regions, W_c = 1.5 μm. Devices correspond to those in **Fig. 1a-c** of the main text, with I_D = 300 μA/μm at V_{DS} = 1 V. Top panels show the temperature distribution map, bottom panels show ΔT along the middle of the channel, dashed line. Heat generation is assumed to be uniform in the channel, and distributed under the contacts with $R_c \approx 1$ kΩ·μm. The power dissipation and ΔT are essentially identical, *i.e.*, the widened W_c does not play an electrical or thermal role. This is largely due to the Au contacts overlapping the nanoribbon channel by $L_{ov} \approx 100$ nm, as in our experimental devices (see main text **Fig. 1a**). The thermal parameters of the simulations are taken from refs.[2,3] and include finite thermal boundary conductance at MoS_2/SiO_2 and MoS_2/Au interfaces. The simulations are insensitive to the 50 nm thick Au contact thermal conductivity in the range 80–120 $Wm^{-1}K^{-1}$, because this is much larger than for the other materials and acts as an efficient heat spreader. The thermal decay length along the nanoribbons is approximately $L_H \approx 78$ nm in both cases.

3. Nanoribbon vs. microribbon monolayer MoS_2 performance comparison

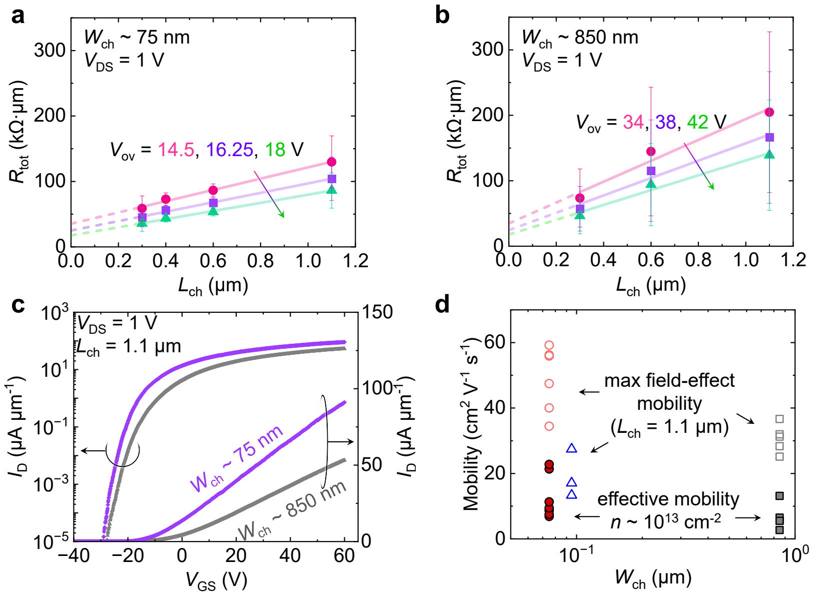

Supplementary Figure 3 | Performance comparison between monolayer MoS_2 nanoribbons and microribbons. Total resistance vs. channel length for **a**, $W_{ch} \approx 75$ nm wide nanoribbons (main text **Fig. 2a-c**) and **b**, wider microribbons on the same chip, with $W_{ch} \approx 850$ nm. Each symbol and error bar represents the average and standard deviation of devices from the same channel length, totaling 30 devices in **a** and 19 devices in **b**. Because W_{ch} of nanoribbons is smaller than the thickness of the back-gate oxide ($t_{ox} \approx 96$ nm here), we account for fringing capacitance using a capacitance per unit area[4] $C_{ox} = \varepsilon_{ox}\left\{\frac{\pi}{\ln[6(t_{ox}/W_{ch}+1)]W_{ch}} + \frac{1}{t_{ox}}\right\}$, where ε_{ox} is the permittivity of SiO_2, and the first term accounts for fringing capacitance while the second term is the typical parallel plate capacitance. This expression reduces to the parallel plate contribution alone when $W_{ch} \gg t_{ox}$. We then estimate the gate-induced carrier density $n \approx C_{ox}(V_{ov} - V_{DS}/2)/q$, where V_{ov} is the gate overdrive ($= V_{GS} - V_T$), V_T is the threshold voltage (estimated at a constant current[1] of $I_D = 0.1$ μA μm^{-1}), and q is the elementary charge. When comparing the total resistance of the nanoribbon vs. microribbon, we find a gate overdrive to match gate-induced carrier densities of 0.8, 0.9, and 1.0 × 10^{13} cm^{-2}. **c**, Measured transfer characteristics of a nanoribbon (purple) and microribbon (gray), here for long-channel devices ($L_{ch} = 1.1$ μm). The same data are shown on log (left) and linear (right) scale. **d**, Estimated mobilities of the nanoribbons vs. microribbons. The field-effect mobility is estimated from long-channel ($L_{ch} = 1.1$ μm) devices, at peak transconductance. The effective mobility is from transfer length method (TLM), estimated at $n \approx 10^{13}$ cm^{-2}. The effective mobility is lower due to the choice of V_T (at constant current) used here. With a linearly extrapolated V_T, the effective mobility would be approximately 20% larger. (See **Supplementary Fig. 5** for additional discussion of uncertainty.)

4. Low-temperature electrical measurements of monolayer MoS_2 nanoribbons on SiO_2

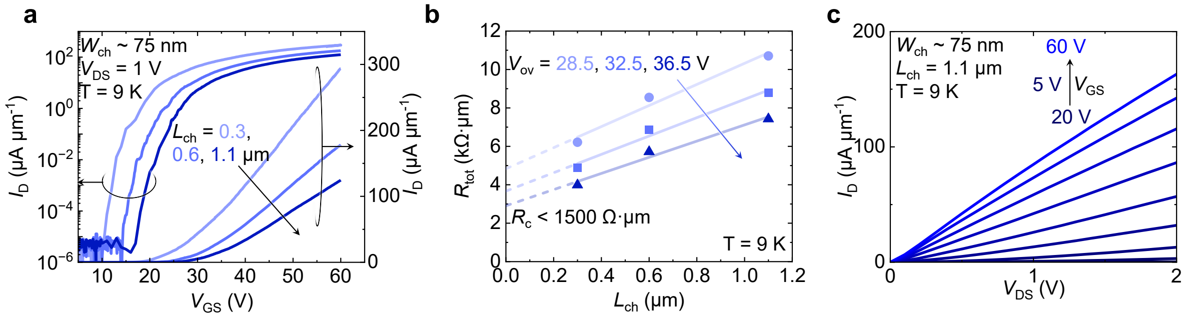

Supplementary Figure 4 | Low temperature measurements of monolayer MoS_2 nanoribbons. a, Transfer characteristics of $W_{ch} \approx 75$ nm wide nanoribbons (main text **Fig. 2a-c**) with varying channel length (L_{ch}, as listed) at $T = 9$ K. **b**, Total resistance vs. channel length for this TLM structure, displaying a contact resistance (R_c) below 1.5 kΩ·µm at the largest gate overdrive. Moreover, we estimate double the mobility (both field-effect and effective) at 9 K compared to room temperature (**Supplementary Fig. 3d**). **c,** Output characteristics of a $W_{ch} \approx 75$ nm wide nanoribbon with $L_{ch} = 1.1$ µm at $T = 9$ K, displaying linear behavior at low bias.

5. Nanoribbon width dependence and hysteresis on multi-patterning (LELE) chip

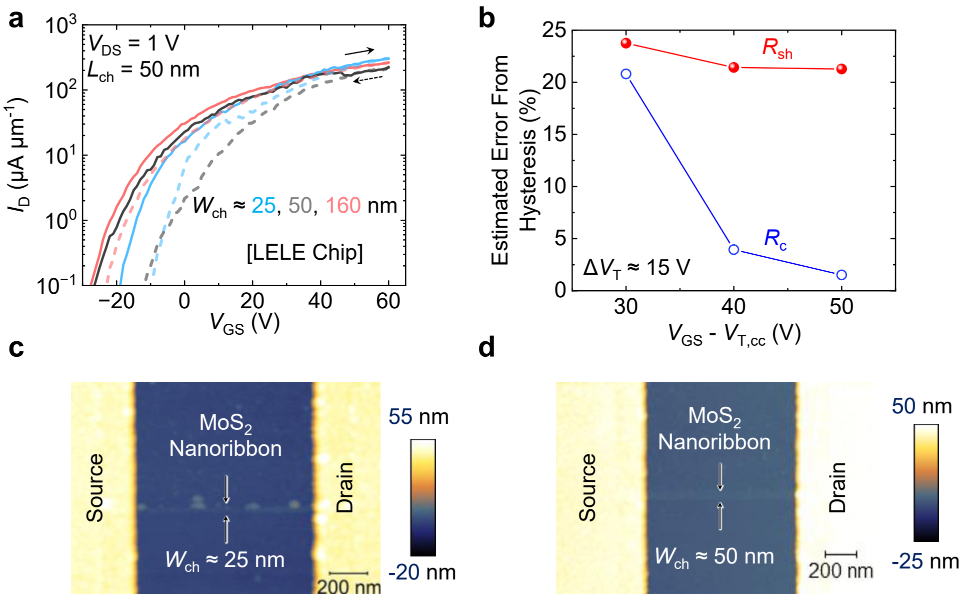

Supplementary Figure 5 | Channel width dependence for monolayer MoS_2 transistors using multi-patterning approach. a, Measured transfer characteristics of nanoribbon devices [main text **Fig. 2d**, multi-patterning or litho-etch-litho-etch (LELE) chip as described in main text **Fig. 1e**] with varying channel width. Here, we do not observe on-state current dependence on the channel width. Instead, signs of variability may arise from contact resistance or monolayer MoS_2 growth variation. Our devices exhibited some clockwise hysteresis[5], as shown in this figure (solid and dashed lines represent forward and backward sweeps respectively). We do not observe a conclusive trend on hysteresis (due to variability) as a function of channel width; however, this hysteresis could impact analysis of R_c and mobility. **b**, Estimated uncertainty for R_c and sheet resistance (R_{sh}) from hysteresis, as a function of gate voltage overdrive, $V_{GS} - V_T$. The estimate is from TLM analysis on a $W_{ch} \approx 50$ nm monolayer MoS_2 channel, where R_c and R_{sh} are calculated from forward and backward sweeps, then the error is their difference divided by their average at the same gate overdrive ($V_{GS} - V_T$). The error from hysteresis reduces at high $V_{GS} - V_T$, as expected, especially for R_c. In other words, hysteresis effects are minimized at high $V_{GS} - V_T \gg \Delta V_T$, rendering conclusions about I_{max} and R_c more robust. The average hysteresis for this structure was $\Delta V_T \approx 15$ V between forward and backward sweeps (at 0.1 μA μm^{-1}), on a 96 nm SiO_2 back-gate dielectric. All transfer curves in this manuscript are the forward sweep, unless otherwise stated. **c**, Atomic force microscopy (AFM) image of 25 nm wide nanoribbon and **d**, AFM of 50 nm wide nanoribbon showing the topography of the devices, both prepared with the multi-patterning approach.

6. Tip enhanced photoluminescence (TEPL) excitation dependence

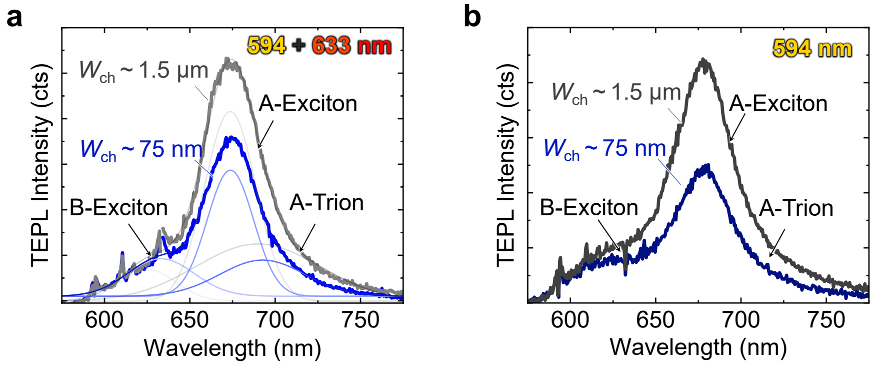

Supplementary Figure 6 | Excitation dependence of tip-enhanced photoluminescence (TEPL) mapping on a 75 nm monolayer MoS_2 nanoribbon. a, Averaged TEPL spectra (thicker line) from **Fig. 3c** in the main text, and corresponding Gaussian peak fittings for the B-exciton, A-exciton, and A-trion (thinner lines). Here, we examine concurrent excitation with both 594 nm and 633 nm lasers. Blue line is taken on the nanoribbon channel ($W_{ch} \approx 75$ nm), gray line is taken for comparison, on a wider region (~1.5 μm). **b**, Averaged TEPL spectra from the same sample, only using the 594 nm excitation laser. We find lower signal-to-noise ratios using only one laser excitation, in addition to a slight change in the peak shapes. The averaging is taken along each respective channel width, from the map in main text **Fig. 3c**.

7. Energy dispersive X-ray spectroscopy (EDS) and electron energy loss spectroscopy (EELS) at a monolayer MoS_2 nanoribbon corner

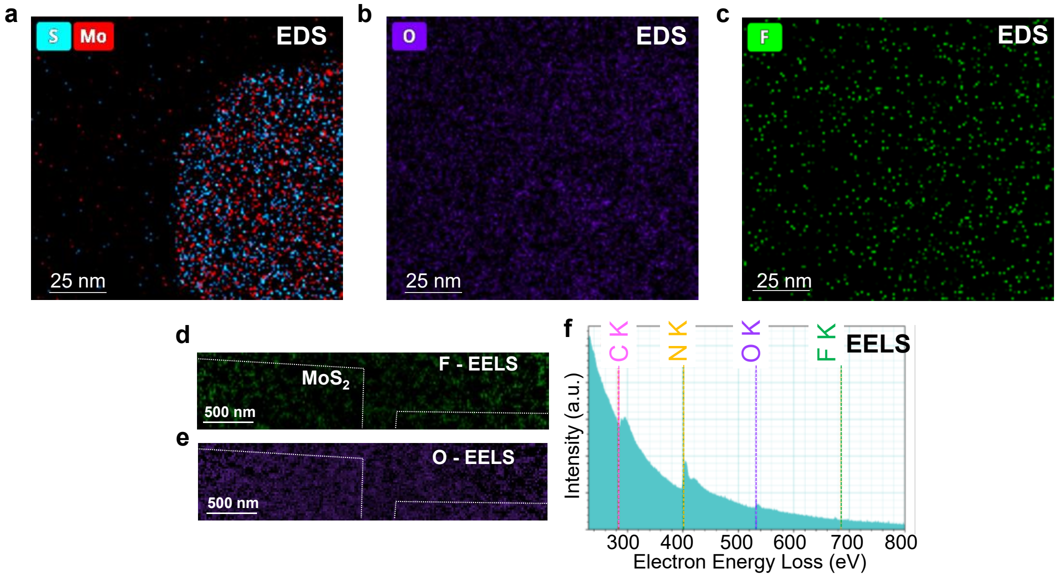

Supplementary Figure 7 | Energy dispersive X-ray spectroscopy (EDS) and electron energy loss spectroscopy (EELS) of a monolayer MoS_2 nanoribbon corners. a, EDS mapping was conducted in conjunction with scanning transmission electron microscopy experiments. Here, we illustrate a map consisting of the Mo and S atoms, indicating the location of the nanoribbon. The black regions are the thin SiN_x supporting membrane. **b**, EDS mapping of O contribution across the same location as (a), indicating no notable O accumulation on the monolayer MoS_2. **c**, We also examine F signal, suggesting no residual influence along the MoS_2 edges from the etching chemistry. EELS is better suited to examine the presence of low-Z (i.e. low atomic number) elements; therefore, we also examine EELS maps of the **d,** F and **e,** O signals close to two MoS_2 edges (indicated using dotted lines). **f,** Spatially averaged EELS spectrum from the entire map in d-e, showing only weak C, N, O, and F signals altogether.

8. TEPL and EELS analysis on monolayer WSe_2

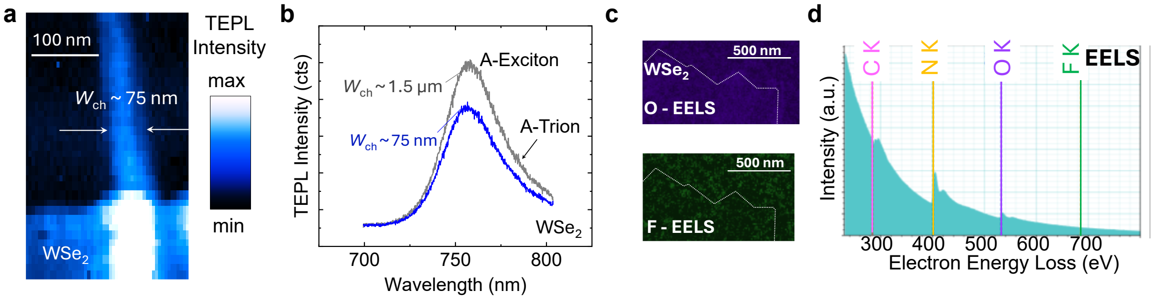

Supplementary Figure 8 | Additional metrology for monolayer WSe_2 nanoribbons and edges. a, TEPL intensity map of a ~75 nm wide WSe_2 nanoribbon, with uniform optical emission along the channel. **b**, Averaged tip-enhanced photoluminescence (TEPL) spectra comparing a nanoribbon channel region to the much wider anchor region of the same WSe_2 nanoribbon, showing minimal broadening of the A-exciton peak and a slight increase in the trion-to-exciton intensity ratio, exactly what is observed for monolayer MoS_2 in **Fig. 3b** in the main text. We note that the TEPL experiments were conducted with both 594 and 633 nm excitation lasers, under the same conditions as **Fig. 3** in the main text. We repeat EELS maps on WSe_2 edges (indicated using dotted lines), displaying the **c,** oxygen and **d,** fluorine signals. **e,** Spatially-averaged EELS spectrum across maps in c-d, showing only weak C, N, O, and F signals.

9. High-angle annular dark-field (HAADF) scanning transmission electron microscopy

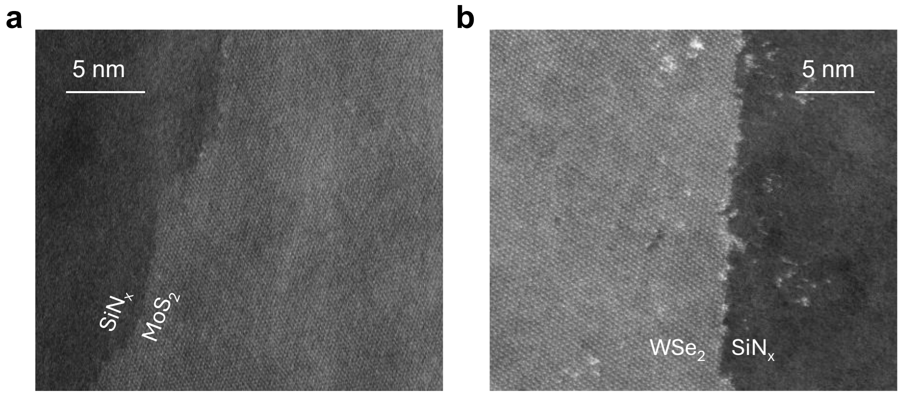

Supplementary Figure 9 | High-angle annular dark-field (HAADF) scanning transmission electron micrograph using an accelerating voltage of 300 kV instead of 80 kV. a, We present another MoS_2 nanoribbon edge, imaged at higher accelerating voltage, demonstrating that the edges are relatively consistent with those presented in **Fig. 3e** of the main text. We note that this imaging configuration is more sensitive to atomic number, thus produces clearer contrast between the monolayer MoS_2 nanoribbon and the SiN_x membrane. Moreover, this nanoribbon edge represents the roughest edge found across the nanoribbon arrays, where the edge roughness here is still a couple of nanometers, as seen in the main text **Fig. 3e**. **b,** HAADF STEM of a monolayer WSe_2 nanoribbon edge, demonstrating that the same processing produces comparable line edge roughness across different TMDs (< 2 nm). The edge quality in these images may be degraded by the layer-transfer process, because the nanoribbons are dry-transferred onto the SiN_x membrane for imaging *after* being patterned (as described in **Methods**). Conversely, for nanoribbon transistors, continuous monolayer films are first grown or transferred onto their substrates *before* being patterned and etched.

10. Micro-Raman spectroscopy on monolayer WS_2 and WSe_2 nanoribbons

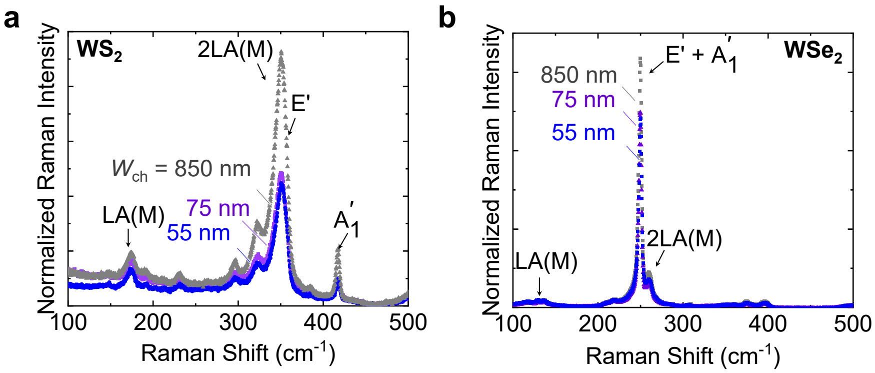

Supplementary Figure 10 | Micro-Raman spectroscopy on monolayer WS_2 and WSe_2 nanoribbons. **a**, Raman spectroscopy on monolayer WS_2 nanoribbons down to 55 nm widths. **b**, Raman spectroscopy on monolayer WSe_2 nanoribbons down to 55 nm widths. We use a 532 nm laser excitation, ~120 µW laser power, and a 1800 l/mm spectrometer grating (same as **Fig. 3a** in the main text). The nanoribbons presented here are from the low equivalent thickness oxide substrates in main text **Fig. 4**, thus continuous monolayer films were transferred onto ~7.5 nm HfO_2 coated SiO_2/Si substrates then patterned into nanoribbons as described in the **Methods** section. We note that the nanoribbons for Raman spectroscopy here were patterned and measured away from the Ti/Pt gate metals. All spectra are normalized to the Si substrate peak at ~520 cm^{-1} (not shown). Importantly, we find that the nanoribbon width does not affect the contribution of defect-mediated peaks[6] [predominantly the LA(M) peaks] across all the 2D monolayers.

11. Output current-voltage characteristics for monolayer MoS_2 nanoribbon on HfO_2

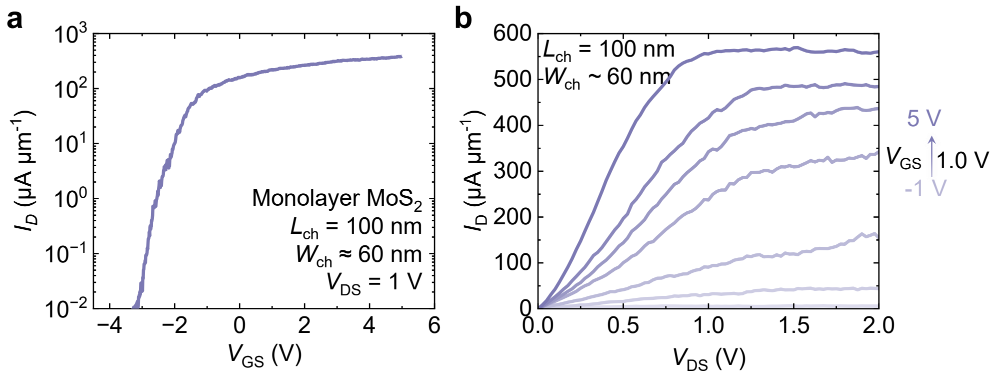

Supplementary Figure 11 | Electrical data of monolayer MoS_2 nanoribbon on thin HfO_2 back-gate dielectric. a, Measured transfer and **b**, output characteristics of the nanoribbon monolayer device on thin HfO_2 (~7.5 nm) back-gate dielectric. The nanoribbon shows current saturation $I_{D,sat} \approx 560$ μA μm^{-1} over a range of voltages $V_{DS} \geq 1$ V, the highest on-state current we observed for monolayer MoS_2 on thin HfO_2 substrates. The device geometry is shown in main text **Figs. 4a-b**. This nanoribbon is slightly wider ($W_{ch} \approx$ 60 nm) than the others shown in **Fig. 4** ($W_{ch} \approx 50$ nm), where all three TMDs are compared.

12. Contact resistance estimate for WS_2 nanoribbons and comparison to microribbons

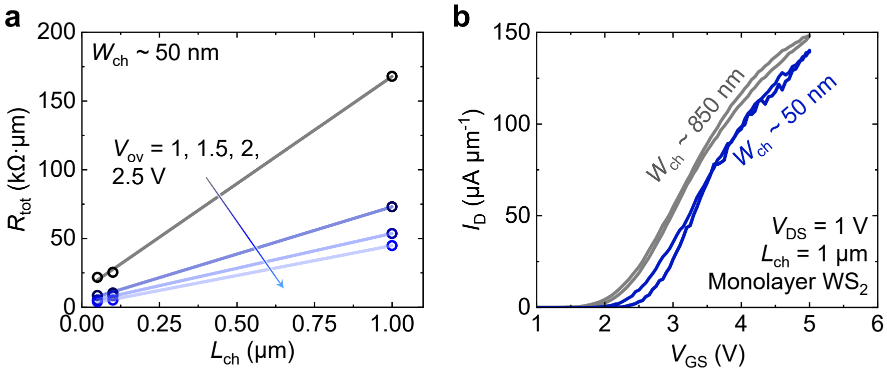

Supplementary Figure 12 | Pseudo transfer length method analysis of monolayer WS_2 back-gated devices. a, Total resistance vs. channel length (mean values across 15 individual devices with different channel lengths[7]), where we estimate R_c = 675 ± 268 Ω·μm at V_{ov} = 2.5 V. (Extrapolated to L_{ch} = 0 and dividing R_{tot} by two.) These devices are back-gated on ~7.5 nm HfO_2, as shown in main text **Fig. 4a-b**. **b**, Transfer characteristics of microribbon vs. nanoribbon monolayer WS_2 with long channels (L_{ch} = 1 μm), demonstrating similar maximum current density, i.e. no indication of mobility or contact resistance degradation, after accounting for the threshold voltage shift, V_T (the two curves are essentially parallel). We also observe slightly increased V_T with reduced channel width for monolayer MoS_2 nanoribbons on HfO_2, see **Extended Data Fig. 4**.

13. Benchmarking Single-Gate Monolayer TMD Semiconductor Nanoribbons

Ref. #	TMD (all *n*-type if not noted)	W_{ch} (nm)	L_{ch} (nm)	$\|V_{DS}\|$ (V)	$\|I_{max}\|$ (μA/μm)	Mobility @ 293 K ($cm^2V^{-1}s^{-1}$)	Gate Dielectric (all single-gated)	Processing Notes
8	MoS_2	30	160	1	2.5	8.5	285 nm SiO_2	Scanning Probe Lithography + XeF_2 etch
9	MoS_2	50	500	0.1	2	/	~21 nm hBN	Electron Beam Lithography + SF_6 etch
10	MoS_2	50	160	1	29	50	300 nm SiO_2	Electron Beam Lithography + SF_6 etch
11	MoS_2	8	1000	1	20	/	SiO_2	Nanoparticle assisted growth
12	MoS_2	40	135	0.75	65	/	50 nm SiO_2 1 nm AlO_x + 10 nm HfO_2 Capping	N/A
13	MoS_2	60	24	0.95	200	/	~8.5 nm HfO_2	Electron Beam Lithography + etch
14	MoS_2	30-40	180	1	485	/	~6 nm HfO_2	Electron Beam Lithography + Cl_2O_2 etch
15	WSe_2 (*p*-type)	56	270	1	57	53	285 nm SiO_2	Scanning Probe Lithography + Passivated WO_x edges
16	WS_2	20 ± 10 (array)*	10^4	1	0.5	/	300 nm SiO_2	Organic nanostructure mask + O_2 plasma etch
**This work**	MoS_2	25	100	1	310	/	96 nm SiO_2	Electron Beam Lithography + XeF_2 etch
	MoS_2	43	300	1	620	/	96 nm SiO_2	〃
	MoS_2	75	300	1	400	58	96 nm SiO_2	〃
	MoS_2	75 (array)	100	1	210	/	96 nm SiO_2	〃
	MoS_2	60	50	1	560	/	~7.5 nm HfO_2	〃
	WS_2	50	50	1	420	/	~7.5 nm HfO_2	〃
	WSe_2 (*p*-type)	50	50	1	130	/	~7.5 nm HfO_2	〃

Table S1: Reported $|I_{max}|$ for single-gated monolayer TMD nanoribbons with W_{ch} < 100 nm. Data are shown at highest $|V_{GS}|$ reported and at the V_{DS} as listed (typically 1 V, sometimes less). All nanoribbons were *n*-type except two WSe_2 reports are *p*-type. All measurements were on single nanoribbons except two marked "array" (*for ref. [16] we estimate 10 nanoribbons in the array). The gate dielectric material and thickness, channel dimensions, and some process details are summarized in the table. Our WS_2 (*n*-type) and WSe_2 (*p*-type) channels in the last two rows represent the highest $|I_{max}|$ reported to date for all monolayer nanoribbons of these materials. All three TMD nanoribbons reported here have the highest $|I_{max}|$ to date among single-gate devices. The nanoribbon I_{max} can be further improved with gate-all-around geometry[17,18].

References

1. Ortiz-Conde, A., García Sánchez, F. J., Liou, J. J., Cerdeira, A., Estrada, M. & Yue, Y. A review of recent MOSFET threshold voltage extraction methods. *Microelectron. Reliab.* **42**, 583–596 (2002).
2. Yalon, E., McClellan, C. J., Smithe, K. K. H., Muñoz Rojo, M., Xu, R. L., Suryavanshi, S. V., Gabourie, A. J., Neumann, C. M., Xiong, F., Farimani, A. B. & Pop, E. Energy Dissipation in Monolayer MoS_2 Electronics. *Nano Lett.* **17**, 3429–3433 (2017).
3. Gabourie, A. J., Köroğlu, Ç. & Pop, E. Substrate-dependence of monolayer MoS_2 thermal conductivity and thermal boundary conductance. *J. Appl. Phys.* **131**, 195103 (2022).
4. Liao, A. D., Wu, J. Z., Wang, X., Tahy, K., Jena, D., Dai, H. & Pop, E. Thermally Limited Current Carrying Ability of Graphene Nanoribbons. *Phys. Rev. Lett.* **106**, 256801 (2011).
5. Urban, F., Giubileo, F., Grillo, A., Iemmo, L., Luongo, G., Passacantando, M., Foller, T., Madauß, L., Pollmann, E., Geller, M. P., Oing, D., Schleberger, M. & Di Bartolomeo, A. Gas dependent hysteresis in MoS_2 field effect transistors. *2D Mater.* **6**, 045049 (2019).
6. Mignuzzi, S., Pollard, A. J., Bonini, N., Brennan, B., Gilmore, I. S., Pimenta, M. A., Richards, D. & Roy, D. Effect of disorder on Raman scattering of single-layer MoS_2. *Phys. Rev. B* **91**, 195411 (2015).
7. Smithe, K. K. H., Suryavanshi, S. V., Muñoz Rojo, M., Tedjarati, A. D. & Pop, E. Low Variability in Synthetic Monolayer MoS_2 Devices. *ACS Nano* **11**, 8456–8463 (2017).
8. Chen, S., Kim, S., Chen, W., Yuan, J., Bashir, R., Lou, J., van der Zande, A. M. & King, W. P. Monolayer MoS_2 Nanoribbon Transistors Fabricated by Scanning Probe Lithography. *Nano Lett.* **19**, 2092–2098 (2019).
9. Jiang, J., Doan, M.-H., Sun, L., Ghimire, M. K., Kim, H., Yun, S. J., Yang, H., Duong, D. L. & Lee, Y. H. Schottky-barrier quantum well in two-dimensional semiconductor nanotransistors. *Mater. Today Phys.* **15**, 100275 (2020).
10. Kotekar-Patil, D., Deng, J., Wong, S. L., Lau, C. S. & Goh, K. E. J. Single layer MoS_2 nanoribbon field effect transistor. *Appl. Phys. Lett.* **114**, 013508 (2019).
11. Li, X., Wyss, S., Yanev, E., Li, Q.-J., Wu, S., Sun, Y., Unocic, R. R., Stage, J., Strasbourg, M., Sassi, L. M., Zhu, Y., Li, J., Yang, Y., Hone, J., Borys, N., Schuck, P. J. & Harutyunyan, A. R. Width-dependent continuous growth of atomically thin quantum nanoribbons from nanoalloy seeds in chalcogen vapor. *Nat. Commun.* **15**, 10080 (2024).
12. Panarella, L., Kaczer, B., Smets, Q., Tyaginov, S., Saraza Canflanca, P., Vici, A., Verreck, D., Schram, T., Lin, D., Knobloch, T., Grasser, T., Lockhart de la Rosa, C., Kar, G. S. & Afanas'ev, V. Evidence of contact-induced variability in industrially-fabricated highly-scaled MoS_2 FETs. *Npj 2D Mater. Appl.* **8**, 44 (2024).
13. O'Brien, K. P., Dorow, C. J., Penumatcha, A., Maxey, K., Lee, S., Naylor, C. H., Hsiao, A., Holybee, B., Rogan, C., Adams, D., Tronic, T., Ma, S., Oni, A., Gupta, A. S., Bristol, R., Clendenning, S., Metz, M. & Avci, U. Advancing 2D Monolayer CMOS Through Contact, Channel and Interface Engineering. in *2021 IEEE International Electron Devices Meeting* (2021). doi:https://doi.org/10.1109/IEDM19574.2021.9720651.
14. Lan, H.-Y., Yang, S.-H., Kantre, K.-A., Cott, D., Tripathi, R., Appenzeller, J. & Chen, Z. Reliability of high-performance monolayer MoS_2 transistors on scaled high-κ HfO_2. *Npj 2D Mater. Appl.* **9**, 5 (2025).
15. Chen, S., Zhang, Y., King, W. P., Bashir, R. & van der Zande, A. M. Edge-Passivated Monolayer WSe_2 Nanoribbon Transistors. *Adv. Mater.* **36**, 2313694 (2024).
16. Aslam, M. A., Tran, T. H., Supina, A., Siri, O., Meunier, V., Watanabe, K., Taniguchi, T., Kralj, M., Teichert, C., Sheremet, E., Rodriguez, R. D. & Matković, A. Single-crystalline nanoribbon network field effect transistors from arbitrary two-dimensional materials. *Npj 2D Mater. Appl.* **6**, 76 (2022).
17. Jaikissoon, M., Buragohain, P., Mortelmans, W., Oguz, K., Rogan, C., Lux, J., Kitamura, A., Engel, C., Vreeland, R. F., Barnett, H., Brooks, Z., Harlson, S., Pinnepalli, S. S. K., Gillispie, E., Toku, K., Wilson, T., Oni, A., Penumatcha, A. V., Dorow, C. J., Kavrik, M., Kozhakhmetov, A., Maxey, K.,

Arefin, N., Kevek, J., Tronic, T., Metz, M., Clendenning, S. B., O'Brien, K. P. & Avci, U. Record PMOS WSe_2 GAA Performance Using Contact Planarization, and Systematic Exploration of Manufacturable, High-Yield Contacts. in *2025 Symposium on VLSI Technology and Circuits* (2025). doi:10.23919/VLSITechnologyandCir65189.2025.11075202.

18. Mortelmans, W., Buragohain, P., Kitamura, A., Dorow, C. J., Rogan, C., Siddiqui, L., Ramamurthy, R., Lux, J., Zhong, T., Harlson, S., Gillispie, E., Wilson, T., Toku, R., Oni, A., Penumatcha, A., Kavrik, M., Jaikissoon, M., Maxey, K., Kozhakhmetov, A., Cheng, C.-Y., Lin, C.-C., Lee, S., Vyatskikh, A., Arefin, N., Kencke, D., Kevek, J., Tronic, T., Metz, M., Clendenning, S. B., O'Brien, K. P. & Avci, U. Gate Oxide Module Development for Scaled GAA 2D FETs Enabling SS<75mV/d and Record I_{dmax}>900μA/μm at L_g<50nm. in *2024 IEEE International Electron Devices Meeting* (2024). doi:https://doi.org/10.1109/IEDM50854.2024.10873417.
